# Is total colectomy for colorectal cancer contraindicated in elderly patients?

**DOI:** 10.1186/1471-2318-11-S1-A24

**Published:** 2011-08-24

**Authors:** S Grassia, C La Tessa, S Spiezia, R Romagnuolo, N Carlomagno, C Dodaro, A Renda

**Affiliations:** 1 Surgical, Anaesthesiology-Rianimative and Emergency Science Department, University of Naples "Federico II", Italy

## Background

More and more frequently elderly patients are operated on for colorectal cancer (CRC). Total colectomy is indicated in selected cases such as synchronous tumors, cancer in FAP, HNPCC, emergency. We evaluated the impact of the age risk factor on patients’ outcome after total colectomy for cancer.

## Methods

We reviewed our series concerning 27 patients submitted to total colectomy for CRC between January 2000 and September 2010. Indications were: synchronous tumors, cancer in FAP, HNPCC, and emergency. We divided them into two groups according to their age: 11 (40.7%) < 65 years (Group A) and 16 (59.3%) ≥ 65 years (Group B). For both groups the following preoperative parameters were investigated: emergency/elective surgery, comorbidity, smoke abuse, ASA score, and surgical operation. Postoperative course related to systemic (pulmonary, cardiac, renal and liver failure, pulmonary embolism, urinary tract infections) and specific surgical complications (anastomotic dehiscence, hemorrhage, postoperative anemia, wound infection, prolonged ileus >3 days) were compared in two groups.

## Results

Comorbidity was higher in older patients. We found a different distribution of ASA scores in the two groups: in the first group 5 (45.5%) patients had score ≤ 2, while 6 (54.5%) patients presented score ≥ 3; in the second group 10 (62.5%) patients presented score 3, while 6 (37.5%) patients had score 4.

We performed 2 (7.4%) total proctocolectomy and permanent ileostomy; 1 (3.7%) restorative proctocolectomy with ileal pouch-anal anastomosis (IPAA); 24 (88.9%) total colectomy and ileo-rectal anastomosis (IRA). We observed 1 hemorrhage in a patient belonging to group A with ASA score 2. Systemic complications were higher in group B. We did not observe significant differences for surgical complications but for postoperative anemia (more frequent in group B). Average hospital stay was similar (11.2 days vs 12.8 days). Mortality [2/27 (7.4%), in group B (2/16-12.5%)] was not statistically significant.

## Conclusions

After total colectomy for CRC we observed slightly higher postoperative morbidity and mortality in elderly patients. However our morbidity, mostly due to systemic complications, was acceptable and depended on a greater presence of preoperative risk factors. In conclusion we believe that, after an accurate preoperative evaluation, the age is not a contraindication for total colectomy.
